# Multiple Culprit Coronary Artery Thrombosis in a Patient with Coronary Ectasia

**DOI:** 10.1155/2018/6148470

**Published:** 2018-01-14

**Authors:** Bruno da Silva Matte, Gustavo Neves de Araujo, Felipe Homem Valle, Ana Maria Rocha Krepsky

**Affiliations:** Hospital de Clínicas de Porto Alegre, Federal University of Rio Grande do Sul, Porto Alegre, RS, Brazil

## Abstract

We here report a case of ST-elevation myocardial infarction (STEMI) due to simultaneous acute coronary artery occlusions of two major coronary arteries in a patient with coronary ectasia. The patient had been previously submitted to percutaneous coronary angioplasty with bare metal stent implantation in both culprit vessels. Very late stent thrombosis could be the cause of the first occlusion, triggering the event in the other vessel. In addition, concomitant embolic sources were not identified. Although routine aspiration thrombectomy in STEMI was not proven to be beneficial in randomized clinical trials, it was of great value in this case. We also discuss the relation between coronary ectasia, chronic inflammatory status, and increased platelet activity which may have caused plaque disruption in another already vulnerable vessel.

## 1. Introduction

Multivessel acute thrombosis leading to ST-segment elevation myocardial infarction is rare, with a potentially devastating clinical presentation due to the vast myocardial area jeopardized [1]. Coronary artery ectasia (CAE) is characterized by abnormal dilatation of a localized or diffuse segment of epicardial coronary arteries, and its presence results in alterations in blood flow and stasis, predisposing to adverse coronary events [2]. On the one hand, current guidelines discourage routine thrombus aspiration techniques [3]. On the other hand, the performance of primary coronary angioplasty in the scenario of both high thrombotic burden and ectasic coronary artery may be facilitated by the adoption of aspirative thrombectomy approach. We present a case of ST-elevation myocardial infarction (STEMI) due to simultaneous thrombotic occlusions of two major coronary arteries in a patient with coronary ectasia, successfully treated with thromboaspiration followed by bare metal stent (BMS) implantation and chronic anticoagulation.

## 2. Case Presentation

A 56-year-old male patient with a history of current tobacco use and previous coronary artery disease was admitted to the emergency room with acute coronary syndrome. He had previous percutaneous coronary intervention (PCI) history in mid left anterior descendent (LAD) and proximal right coronary artery (RCA) with bare metal stents in a context of unstable angina two years before in another facility and was on aspirin 100 mg daily. Clopidogrel had been used for one year and then discontinued. He arrived at the hospital within five hours and thirty minutes from the onset of severe chest pain associated with nausea and diaphoresis. His blood pressure was 130/80 mmHg, heart rate was 80 bpm, cardiac auscultation was unremarkable, and his Killip class was II.

**Figure 1:**
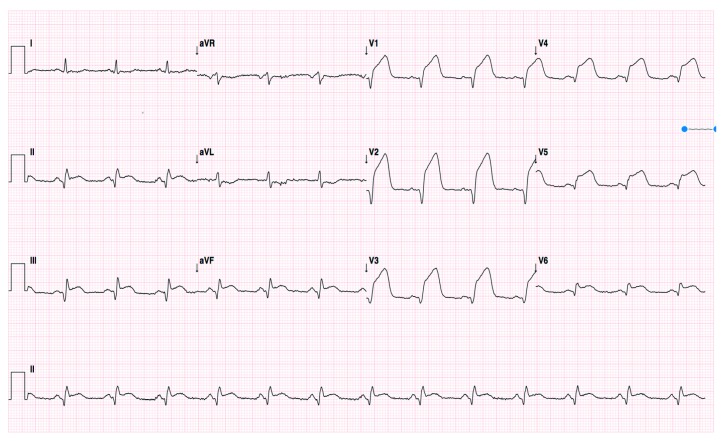
Admission electrocardiogram.

**Figure 2:**
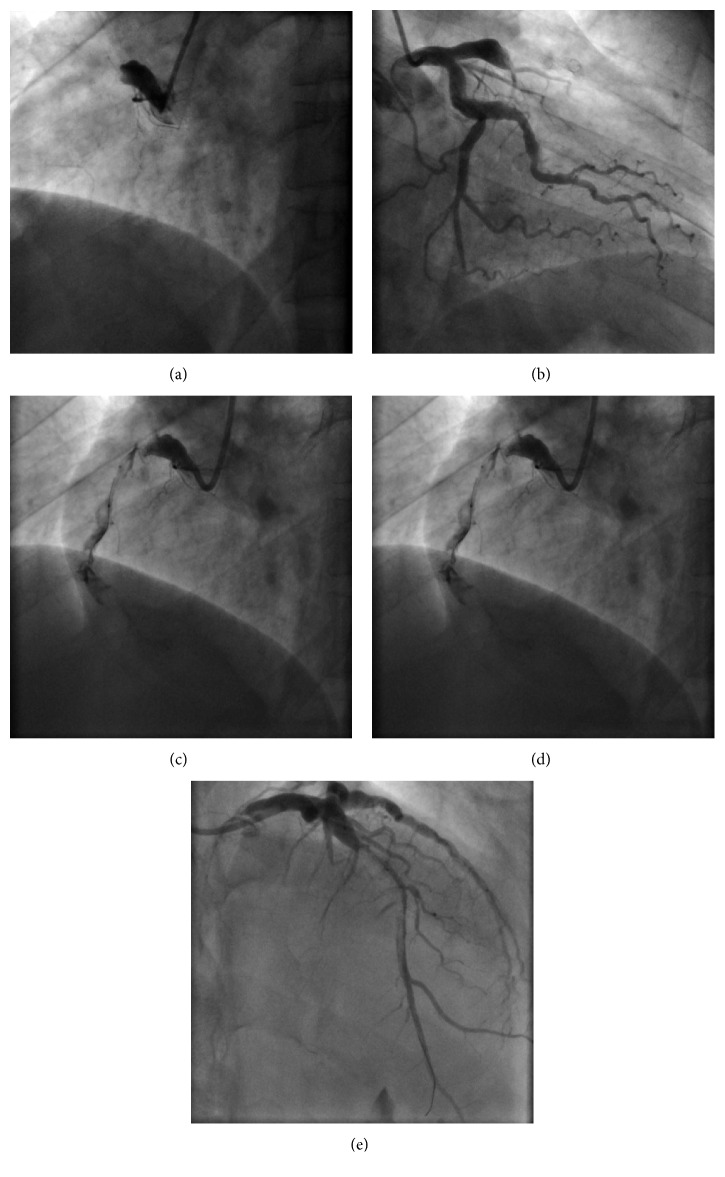
Coronary angiography. Marked ectasia and proximal occlusion of right coronary artery (a) and left anterior descending coronary artery (b). After initial ballon angioplasty, both vessels showed extensive thrombus burden and flow restriction (c and d).

Admission ECG showed sinus rhythm with both anterior and inferior ST-segment elevation (Figure 1). Oral loading doses of antiplatelets (aspirin 300 mg and clopidogrel 600 mg) were administered, and coronary angiography was performed emergently. Surprisingly, thrombotic occlusions of both proximal RCA (Figure 2(a)) and mid LAD (Figure 2(b)) were disclosed. Unfractionated heparin (100 UI/kg) and IIb/IIIa glycoprotein inhibitor (abciximab) were administered during the procedure, and the latter was continued up to 12 hours after the procedure. Both vessels were diffusely ectasic, and the occlusions occurred into pronounced ectasic spots. LAD thrombosis occurred just proximal to the previously implanted stent, and RCA thrombosis occurred in the proximal segment while the previously implanted stent was on the distal segment. There were only discrete collaterals from LAD to RCA after the first vessel was recanalized.

**Figure 3:**
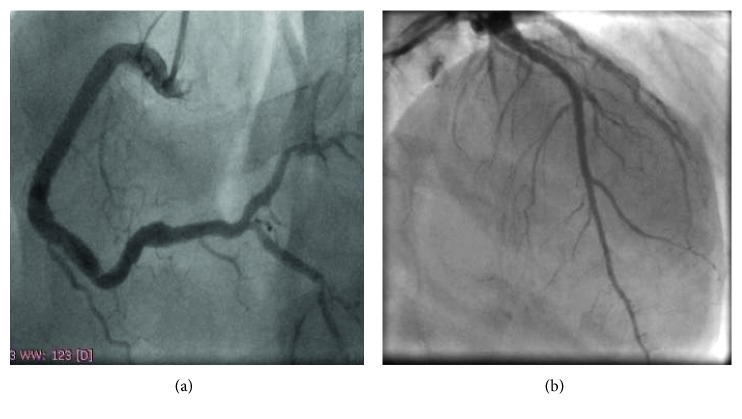
Final angiographic result. Both right coronary artery (a) and left anterior descending coronary artery (b) showing good angiographic result, with TIMI 3 flow and no residual stenoses.

We decided to intervene first on LAD due to the larger myocardium area at risk. Guidewire easily surpassed the occluded segment, and LAD was predilated with 2.0 × 15 and 2.5 × 20 mm noncompliant balloons (NCB) without flow restoration despite several inflations. Manual thrombus aspiration with Export catheter was performed, and such approach allowed much better thrombus clearance and vessel delineation (Figure 2(d)). Kaname 3.0 × 28 mm BMS was then implanted and postdilated with a 3.5 × 15 mm NCB. RCA intervention was performed in the same procedure, and the vessel had a similar behavior, with easy guidewire navigation and no flow restoration with balloon angioplasty (2.5 × 20 mm and 3.0 × 20 mm). Manual aspiration also allowed better vessel delineation (Figure 2(c)). Multilink 4.0 × 33 mm and Prokinetic 4.0 × 20 mm BMS were implanted in distal and medial segments, respectively, with overlapping and was postdilated with a 5.0 × 20 mm NCB. Angiographic success (Figure 3) was achieved, and IIb/IIIa glycoprotein inhibitor was maintained for 12 hours in continuous infusion.

The patient was in sinus rhythm throughout hospitalization, and echocardiogram showed an ejection fraction of 34% with apical and septal akinesia. It did not show intracardiac thrombus or vegetation, making coronary embolization improbable as a cause of thrombosis. Outpatient hematologic evaluation was scheduled to investigate for possible thrombophilia. After a successful in-hospital evolution, the patient was discharged asymptomatic within six days. Discharge medications were aspirin 100 mg, metoprolol 50 mg BID, enalapril 10 mg BID, simvastatin 40 mg, clopidogrel 75 mg, and warfarin 5 mg. It was planned to use triple therapy for one month and then switch to dual therapy with clopidogrel and warfarin, indefinitely.

## 3. Discussion

Coronary artery ectasia (CAE) is characterized by abnormal dilatation of a localized or diffuse segment of the epicardial coronary arteries with a luminal diameter of at least 1.5-fold of normal adjacent segment or vessel diameter [4]. The incidence of CAE in patients investigated for coronary artery disease is between 1% and 2–5% [5]. It is caused by destruction of the vessel media and frequently coexists with coronary atherosclerosis. Even in the absence of obstructive disease, the presence of dilated coronary segments results in alterations in blood flow and stasis, predisposing to adverse coronary events like vasospasm, thrombosis, and dissection [4]. Recent study by Doi et al. showed higher incidence of major cardiac events, cardiovascular mortality, and nonfatal myocardial infarction in patients with CAE [6].

ST-segment elevation myocardial infarction caused by multiple culprit coronary arteries is rare, with the largest series of cases reporting 47 patients with this condition [7]. Clinical presentation can be devastating because of the vast myocardial area jeopardized, with a high rate of harmful outcomes such as ventricular arrhythmia, severe heart failure, and cardiogenic shock [5]. There are several etiologies of multivessel coronary thrombosis, all of them related to the Virchow's triad which include endothelial dysfunction, hemodynamic changes (i.e., stasis and turbulence), and hypercoagulability [7–9]. Factors probably involved with multiple coronary artery thrombosis in our patient include a proinflammatory state and blood turbulence caused by coronary ectasia, and heightened inflammatory response caused by the acute occlusion of the first vessel.

There are some considerations regarding the management of our patient that needs to be highlighted: First, the choice of the first vessel to be treated was made on the larger myocardium supplied by LAD, and hemodynamic collapse could have occurred if RCA had been treated first due to large delay that would have happened because of complexity of the RCA PCI. It is intriguing that cardiogenic shock did not occur in this case, possibly because of collateral recruitment from the previous coronary events. Second, the combination of coronary ectasia and high thrombus burden, such as in our patient, illustrates a potential scenario of clinical benefit of aspiration thrombectomy. Third, IIb/IIIa glycoprotein inhibitor therapy promptly initiated when anatomy was defined by angiography probably was of great value. Fourth, bare metal stenting was performed aiming chronic anticoagulation, allowing the use of triple antiplatelet therapy for a shorter period and reducing the risk of bleeding. Taken all together, the anatomical and technical issues in this case were very enlightening.
